# Evolutionary implications of the divergent long bone histologies of *Nothosauru*s and *Pistosaurus* (Sauropterygia, Triassic)

**DOI:** 10.1186/1471-2148-13-123

**Published:** 2013-06-18

**Authors:** Anna Krahl, Nicole Klein, P Martin Sander

**Affiliations:** 1 Division of Paleontology, Steinmann Institute, University of Bonn, Nussallee 8, 53115 Bonn, Germany

## Abstract

**Background:**

Eosauropterygians consist of two major clades, the Nothosauroidea of the Tethysian Middle Triassic (e.g., *Nothosaurus*) and the Pistosauroidea. The Pistosauroidea include rare Triassic forms (Pistosauridae) and the Plesiosauria of the Jurassic and Cretaceous. Long bones of *Nothosaurus* and *Pistosaurus* from the Muschelkalk (Middle Triassic) of Germany and France and a femur of the Lower Jurassic *Plesiosaurus dolichodeirus* were studied histologically and microanatomically to understand the evolution of locomotory adaptations, patterns of growth and life history in these two lineages.

**Results:**

We found that the cortex of adult *Nothosaurus* long bones consists of lamellar zonal bone. Large Upper Muschelkalk humeri of large-bodied *Nothosaurus mirabilis* and *N*. *giganteus* differ from the small Lower Muschelkalk (*Nothosaurus marchicus*/*N*. *winterswijkensis*) humeri by a striking microanatomical specialization for aquatic tetrapods: the medullary cavity is much enlarged and the cortex is reduced to a few millimeters in thickness. Unexpectedly, the humeri of *Pistosaurus* consist of continuously deposited, radially vascularized fibrolamellar bone tissue like in the *Plesiosaurus* sample. *Plesiosaurus* shows intense Haversian remodeling, which has never been described in Triassic sauropterygians.

**Conclusions:**

The generally lamellar zonal bone tissue of nothosaur long bones indicates a low growth rate and suggests a low basal metabolic rate. The large triangular cross section of large-bodied *Nothosaurus* from the Upper Muschelkalk with their large medullary region evolved to withstand high bending loads. *Nothosaurus* humerus morphology and microanatomy indicates the evolution of paraxial front limb propulsion in the Middle Triassic, well before its convergent evolution in the Plesiosauria in the latest Triassic. Fibrolamellar bone tissue, as found in *Pistosaurus* and *Plesiosaurus*, suggests a high growth rate and basal metabolic rate. The presence of fibrolamellar bone tissue in *Pistosaurus* suggests that these features had already evolved in the Pistosauroidea by the Middle Triassic, well before the plesiosaurs radiated. Together with a relatively large body size, a high basal metabolic rate probably was the key to the invasion of the Pistosauroidea of the pelagic habitat in the Middle Triassic and the success of the Plesiosauria in the Jurassic and Cretaceous.

## Background

### Sauropterygian phylogeny and evolution of aquatic locomotion

**Figure 1:**
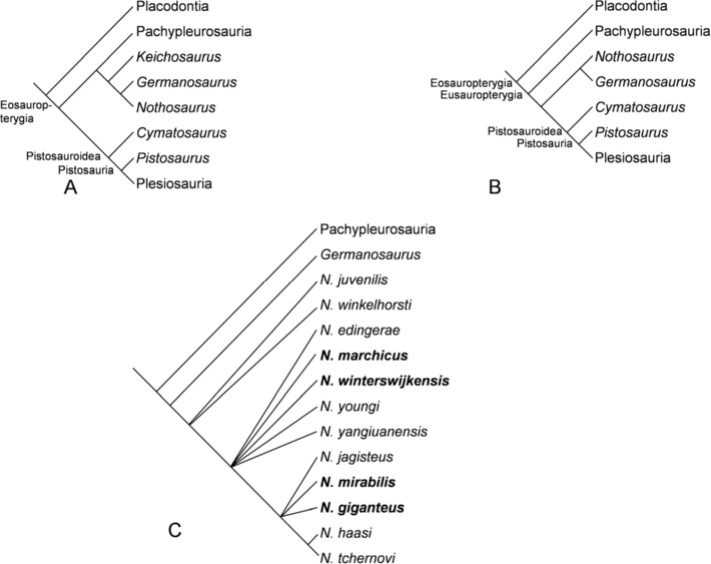
**Contrasting phylogenetic hypotheses of stem-group Sauropterygia and relationships of *Nothosaurus*. A**, In the analysis of Holmes et al. [6], the putative pachypleurosaur *Keichosaurus* plots as a basal nothosauroid, and Pachypleurosauria and Nothosauroidea form a monophyletic taxon, resulting in the loss of the node Eusauropterygia; **B**, The classical view [4] with the Pachypleurosauria as the sister-group of Eusauropterygia including Nothosauroidea and Pistosauroidea; **C**, Phylogeny of the Nothosauridae [modified from 16] with the sampled species indicated in bold. *Nothosaurus winterswijkensis* and *N*. *marchicus* are more basal species than *N*. *giganteus*. *N*. *mirabilis* is one of the most derived nothosaur species.

Since the beginning of the 19th century, the Mesozoic marine reptile clade Sauropterygia has been subject to intensive morphological studies [e.g., [1,2]]. Sauropterygia are divided into the Placodontia and the Eosauropterygia which, in turn, classically consist of the Eusauropterygia and the Pachypleurosauria [3-5] (see also Figure 1). Eusauropterygia are divided into the plesiosaur lineage (Pistosauroidea) and the nothosaur lineage (Nothosauroidea). However, in a recent analysis by Holmes et al. [6], Eosauropterygia remained unresolved in a polytomy, and Eusauropterygia turned out to be paraphyletic (Figure 1A). The Pistosauroidea include the plesiosaurs.

The non-plesiosaurian sauropterygians, commonly referred to as “stem-group sauropterygians”, are known from various localities in Europe [e.g., [7-10]], China [e.g., [6,11]], USA [e.g., [12]], Tunisia [13], and Israel [14]. In the fossil record, they appear for the first time in the latest Early Triassic and disappear in the latest Triassic [4]. The Plesiosauria, also termed “crown-group sauropterygians”, first occur in the earliest Jurassic (or possibly latest Triassic) of Europe [1], but they spread rapidly around the world during the later Jurassic and Cretaceous to become the most diverse group of marine reptiles [15].

One of the most familiar stem-group sauropterygians is *Nothosaurus*, several species of which inhabited the Muschelkalk Sea of the Germanic Basin from early Anisian to early Carnian times (for about 15 Ma). Finds come from the Lower Muschelkalk to the Lower Keuper beds of France, Germany, Poland, The Netherlands, and Switzerland [4]. Unfortunately, phylogenetic relationships within the genus *Nothosaurus* remain largely unresolved (see most recent phylogenetic analysis [16], Figure 1C).

Important stem-group sauropterygians of the plesiosaur lineage are *Cymatosaurus* and *Pistosaurus* from the Muschelkalk beds. While *Cymatosaurus* (total length <1.5 m) appears to be restricted to the Lower Muschelkalk [17], *Pistosaurus* is only found in beds recording maximum flooding of the Germanic Basin in the Upper Muschelkalk [17]. *Pistosaurus* and other Pistosauridae such as *Augustasaurus* from the Anisian of Nevada [12,18] and *Yunguisaurus* from the Carnian of China [11] share a seemingly pelagic lifestyle, consistent with their Northern Hemisphere and presumably cosmopolitan distribution. Pistosauridae are substantially larger (2–3 m total lengh) than the more basal Pistosauroidea such as *Cymatosaurus*[4] and *Corosaurus*[19-21] but are of the same size as the basal Plesiosauria [1].

**Figure 2:**
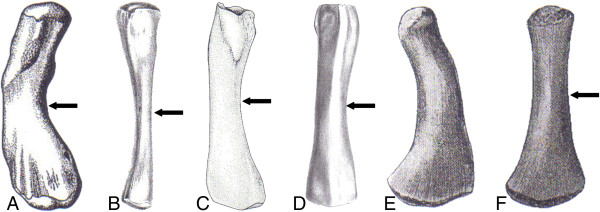
**Sauropterygian long bones with planes of section marked (arrows). A**, humerus of *Nothosaurus* sp. indet. [from [93]]. Note that *Nothosaurus* humeri are morphologically more differentiated than those of the more advanced taxa. **B**, femur of *Nothosaurus* sp. indet. [from [2]]; **C**, humerus of *Pistosaurus longaevus* [from [8]]; **D**, femur of *Pistosaurus longaevus* [from [2]]; **E**, humerus of a plesiosaur [from [94]], figured for better morphological comparison with other sauropterygian long bones; **F**, femur of a plesiosaur [from [94]]. Proximal is at the top in all bones. Bones in A and D-F are shown in dorsal view; bones in B and C are shown in ventral view. Not to scale.

The diverse humerus morphologies of Sauropterygia (Figure 2 A-E) partially reflect adaptations to an increasingly pelagic habitat and also different modes of locomotion, which fundamentally changed from basal Pistosauroidea to Plesiosauria [17,22-26]. While the former mainly employed lateral undulation for propulsion, the latter reduced the tail and uniquely evolved two morphologically nearly identical pairs of flippers, which were employed in a paraxial swimming mode [see [24] for review].

Locomotion in Nothosauroidea clearly differed from either the more basal sauropterygians such as pachypleurosaurs and the basal pistosauroids or plesiosaurs. By reconstructing musculature and based on morphological observations of the pectoral girdle, Carroll and Gaskill [22], Watson [25], and v. Huene [26] hypothesized that Nothosauroidea independently evolved paraxial locomotion, where the front limbs were employed in a “rowing flight”, combining lift- and drag-based elements of propulsion, like recent sea lions [27], while the hind limbs were used for maneuvering.

### Tetrapod long bone histology and secondary aquatic adaptation

Pachyostosis, osteosclerosis, pachyosteosclerosis, and the retention and subsequent calcification of cartilage at distance from the epiphyses are all mechanisms through which secondarily aquatic tetrapods increase their specific density to counteract the positive buoyancy caused by the lungs [28-30]. Pachyostosis, osteosclerosis, and pachyosteosclerosis, subsumed under the term bone mass increase (BMI, [30]), are different ways of increasing bone mass. Pachyostosis is the hyperplasy of bone deposition, leading to a swollen appearance of the bone, and osteosclerosis is the increase in inner bone compactness by an inhibition of cortical resorption and/or excessive deposition of secondary bone [29,30]; pachyosteosclerosis is the combination of pachyostosis and osteosclerosis [29,30]. An increase in body density seems to be typical for vertebrates living in a lagoonal or shallow water habitat, either marine or freshwater [31], like Sirenia [32] and pachypleurosaurs [e.g., [4,22,33].

BMI appears to be the initial step in secondary adaptation to an aquatic environment [29,34]. Further aquatic adaptation results in the reduction in body density through an osteoporotic-like state of the skeleton (sensu [20,34]) because a decrease in body density allows faster acceleration and increased maneuverability [30,34]. An osteoporotic-like state is found in pelagic animals like some modern whales (e.g., [28,30,34,35]), ichthyosaurs (e.g., [30,34,36,37]) and some adult plesiosaurs [30,34,38,39]. While the terminology suggests that the decrease in bone density is brought about by increased cortical resorption activity [30,34], an alternative pathway to bone density reduction would be a strong increase in vascularization, leading to the periosteal bone being primarily cancellous. In either case, decrease in bone density is the terminal state in histological adaptation to a secondarily aquatic mode of life. This histological adaptation does not appear to have been modified further in any secondarily aquatic tetrapod, and there is no known case of a high-performance, pelagic swimmer reinvading shallow-water habitats.

### Biological and histological ontogenetic stages

For non-mammalian fossil tetrapods, bone histology is the most reliable means of ascertaining their ontogenetic stage [40]. The general approach is to correlate specifics of histology that change with ontogeny to life history events. In large samples covering closely related taxa, such specifics of histology and their ontogenic change can be formalized as histologic ontogenetic stages [41].

Life history events that are potentially reflected in bone histology are hatching or birth, sexual maturity, attainment of full size, and senescence [41]. Biological ontogenetic stages are preceded or succeded by these events. Thus, an embryo or fetus exists before hatching or birth; a hatchling or neonate lives after birth; a juvenile lives well past hatching or birth but is not yet approaching sexual maturity; a subadult is approaching sexual maturity, which is recorded in histology by a distinctive slow-down in growth; an adult is sexually mature but not necessarily fully grown; and a senescent individual lives on after having attained final size, attainment of final size being indicated histologically by an external fundamental system (EFS).

In particular, the definition of “adult” has historically led to confusion because of the derived mammalian and avian condition in which sexual maturity coincides with full size (most mammals) or even postdates attainment of full size (birds) [42]. However, the plesiomorphic condition for reptiles (seen in all extant non-avian reptiles) is that sexual maturity is attained well before full size [42], and it is most parsimonious to assume this applied to sauropterygians as well. Thus, the marked decrease in growth rate observable in medium-sized individuals is here interpreted as indicative of sexual maturity (see also [43,44]).

### History of research on sauropterygian histology and microanatomy

Already in the late 19th and early 20th century, the bone histology of sauropterygians was described and illustrated [45-50], sometimes in great detail but with a limited understanding of its meaning. In the modern studies by Enlow and Brown [51] and particularly Ricqlès [36,52], the histology of various reptile taxa, including eosauropterygians, was reviewed, and questions about the function of different bone tissue types and their implications for the thermal regime and for phylogenies of extinct species were raised. Sander [33,53] investigated longevity and life history of the pachypleurosaur *Neusticosaurus* by applying skeletochronology. Ricqlès [54], Ricqlès and Buffrénil [34], and Houssaye [30] discussed various microanatomical adaptations of secondarily aquatic tetrapods to the marine environment connected to different habitats and locomotory styles (including Eosauropterygia). Several recent studies illustrate aspects of plesiosaur bone histology [38,55-58], but only that of Wiffen et al. [38] offers sufficent data to be of use in a broader comparative context. Bone histology of Pistosauridae has not previously been studied.

The most recent studies dealing extensively with stem-group sauropterygian long bone histology are that by Klein [59] and Hugi et al. [60]. Klein [59] surveyed histological diversity of Lower Muschelkalk (Anisian) taxa. Among these, *Nothosaurus* species differ histologically from pachypleurosaurs (*Anarosaurus*) and cf. *Cymatosaurus*. Hugi et al. [60] focussed on Southern Alpine pachypleurosaurs of the genera *Serpianosaurus* and *Neusticosaurus* and found that all show lamellar zonal bone tissue (LZB) with high compactness values.

In this study, we histologically sampled an array of *Nothosaurus* species from the Lower Muschelkalk and from the Upper Muschelkalk to the Lower Keuper beds, *Pistosaurus longaevus* from the Upper Muschelkalk beds, and *Plesiosaurus dolichodeirus* from the Lower Jurassic of Lyme Regis, England [9]. We found a remarkable diversity in histologies among the different *Nothosaurus* species, *Pistosaurus,* and *Plesiosaurus*, reflecting different biomechanical and physiological specializations. We interpret these results in terms of the evolution of locomotory styles and aquatic adaptation.

## Material and methods

### Material

The material examined in this study is mainly from the Middle Triassic Upper Muschelkalk beds and the Grenzbonebed horizon (Lower Keuper) and is thus of Ladinian age (242–235 million years ago, [61]). Geographically it hails from various localities in southern Germany and France (Additional file 1). The Upper Muschelkalk/Grenzbonebed sample comprises complete or partial long bones, which pertain to *Nothosaurus giganteus*, *N*. *mirabilis*, and *Pistosaurus longaevus*. *Nothosaurus mirabilis* and *N*. *giganteus* material had been identified to species level in the collections it came from. Since there is little to no association between the name-bearing cranial material of the species of *Nothosaurus* and postcranial parts of the skeleton, these identifications are based on the dimensions of the bones (Additional file 1). We also sampled two small humeri and one femur that could not be assigned to species to test whether these pertain to juveniles of the larger species or to adults of a species of smaller body size such as *N*. *juvenilis*, *N*. *edingerae*, or *N*. *jagisteus*, all of which are from the Upper Muschelkalk or the Lower Keuper beds. For phylogenetic and stratigraphic comparison (Table 1), a *Nothosaurus* humerus from the Lower Muschelkalk of Förderstedt, south of Magdeburg, Saxony-Anhalt, Germany, was sampled. It belongs to morphotype II of Bickelmann and Sander [62] and can thus be assigned to *Nothosaurus marchicus*/*N*. *winterswijkensis*[62]. *Pistosaurus longaevus* material (Figure 2, Table 1) was assigned to genus and species by morphological and histological comparison with *Pistosaurus* material in the collections of SMNS, and by comparison to the description of *Pistosaurus* by Sues [8]. The plesiosaur specimen (Figure 2, Table 1) is a *Plesiosaurus dolichodeirus* femur in the SIPB collections from the Lower Jurassic of Lyme Regis, U.K., which is either Hettangian or Pliensbachian in age [9].

**Table 1:** Results of the histological examination of eosauropterygian taxa

**Taxon**	**Collection number**	**Bone type**	**Type of section**	**Bone tissue type**	**Number of growth cycles**	**Ontogenetic stage**	**Medullary Index (%)**	**Histological specialization**
*N*. *marchicus*/ *N*. *winterswijkensis*	MfN R 174-2	Humerus	ts of proximal shaft	lzb	7	Adult	42.5	Some osteosclerosis
*N*. *mirabilis*	SIPB R 54/2	Humerus	dts, lts of proximal head, 9 ps	lzb	10	Adult	63.8	Much reduced cortex
*N*. *mirabilis*	SIPB R 50/2	Femur	dts	lzb	9	Subadult	63.0	None
*N*. *mirabilis*	SIPB R 54/1	Femur	dts, lts of proximal head, 8 ps	lzb	8	Adult	42.5	Some osteosclerosis
*N*. *mirabilis*	SIPB R 50/1	Femur	dts	lzb	9	Subadult	32.9	Osteosclerosis
*N*. *giganteus*	SIPB R 45	Humerus	dts	lzb	EFS	Fully grown	58.8	Much reduced cortex
*N*. *giganteus*	SIPB R 53	Humerus	dts	lzb	EFS	Fully grown	77.4	Much reduced cortex
*N*. *giganteus*	SIPB R 40	Humerus	ts of proximal shaft	lzb	/	Adult	74.7	Much reduced cortex
*N*. *giganteus*	MHI 1903	Humerus	2 mts	lzb	8	/	/	/
*N*. *giganteus*	SIPB R 49	Femur	dts	lzb	7	Subadult	75.8	Much reduced cortex
*Nothosaurus sp*. indet.	MHI 1906	Humerus	dts	lzb	3	Juvenile	35.0	Osteosclerosis
*Nothosaurus sp*. indet.	MHI 633	Humerus	dts	flb	3	Juvenile	26.9	Thick cortex of flb
*Nothosaurus sp*. indet.	SMNS 84856	Femur	dts	lzb	10	Adult	27.8	Osteosclerosis
*Pistosaurus longaevus*	SIPB R 46	Humerus	dts	flb	5	Adult	6.7	Thick cortex of flb
*Pistosaurus longaevus*	SMNS 84825	Humerus	dts	flb	7	Adult	5.0	Thick cortex of flb
*Pistosaurus longaevus*	SIPB R 74	Femur	dts	lzb	8	Subadult or adult	65.8	Some osteosclerosis
*Plesiosaurus dolichodeirus*	SIPB R 90	Femur	dts	flb	EFS	Fully grown	35.0	Haversian remodeling

Overview of the sampled taxa, long bones, and histological observations. A low MI means a small medullary region. Abbreviations: *dts* diaphyseal thin section, *EFS* external fundamental system, *flb* fibrolamellar bone, *lts* longitudinal thin section, *lzb* lamellar zonal bone, *mts* metaphyseal thin section, *ps* polished sections, *ts* thin section, */ absent* or growth marks not identifiable.

## Methods

Based on principles of long bone growth [63], the middle region of the shaft at its narrowest part is the best region to sample long bones, because it yields the longest growth record and it corresponds approximately to the neutral zone of growth [64,65]. Skeletochronology, i.e., study of growth marks in bone, does not only provide information on the age of an individual but also on other aspects of its life history, e.g., sexual maturity, bone growth rates, and reproduction cycles [60,64-69]. The terminology used to describe bone histology follows Francillon-Vieillot et al. [64].

**Figure 3:**
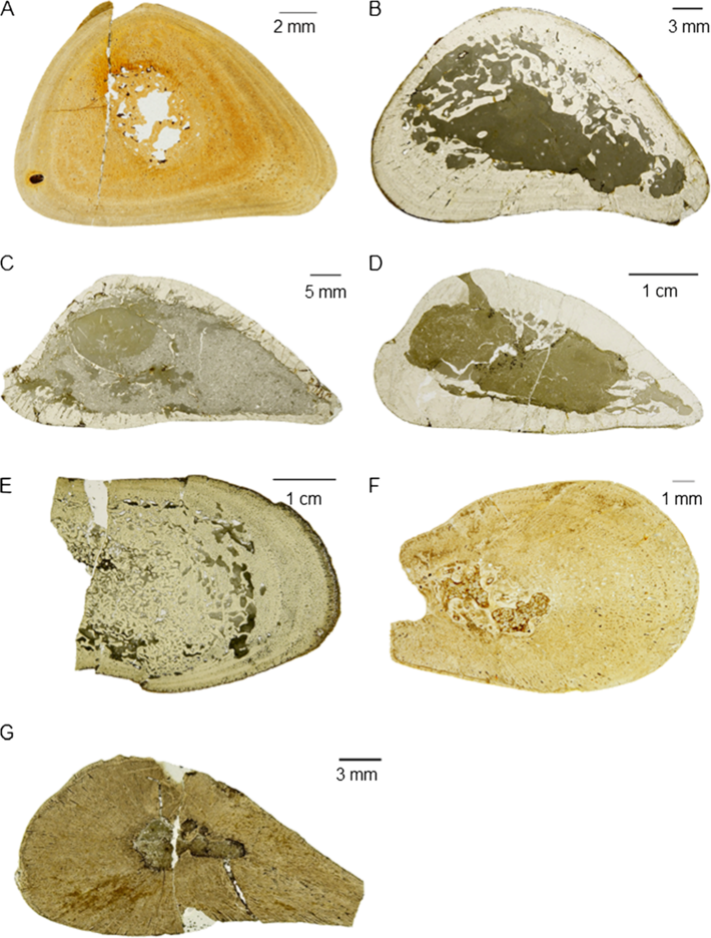
**Overview photographs of *Nothosaurus *humeri thin sections in normal light. A-D** and **F-G**, diaphyseal sections; **E**, metaphyseal thin section. In all images, ventral is at the bottom. **A**, *N*. *marchicus*/*N*. *winterswijkensis* humerus (MfN R 174–2); **B**, *N*. *mirabilis* humerus (SIPB R 54/2); **C-E**, *N*. *giganteus* humeri (**C**, SIPB R 53, **D**, SIPB R 45, **E**, MHI 1903, Section 1); **F-G**, *Nothosaurus*. sp. indet. humeri (**F**, MHI 1906, **G**, MHI 633). Note the triangular cross sections of the *Nothosaurus* humeri (**A-D, F-G**). Humeri in A and F are osteosclerotic (they have a low MI and the medullary regions are mostly infilled by endosteal bone and calcified cartilage), unlike the humeri in B, C and D which show thinner cortices and large medullary regions. The juvenile humerus in G has a small medullary cavity but highly vascularized cortical bone.

**Figure 4:**
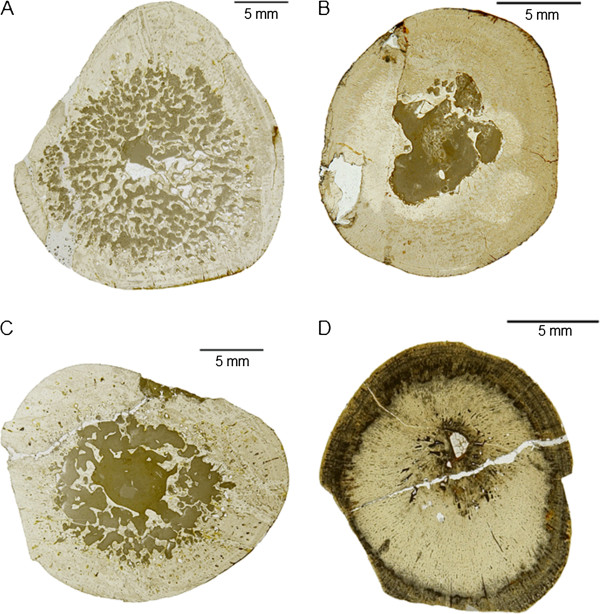
**Overview photographs of *Nothosaurus *femur thin sections in normal light. A-D**, diaphyseal sections. In all images, ventral is at the bottom. **A**, *N*. *giganteus* femur (SIPB R 49); **B**-**C**, *Nothosaurus mirabilis* femora (SIPB R 54/1, SIPB R 50/2); **D**, *Nothosaurus* sp. indet. femur (SMNS 84856). In contrast to the triangular *Nothosaurus* humeri, femora have approximately round cross sections. In the femora in A and C, strong remodeling and a tendency for expansion of the medullary region are visible. The femur in D is clearly osteosclerotic, and the one in B also shows some osteosclerosis.

**Figure 5:**
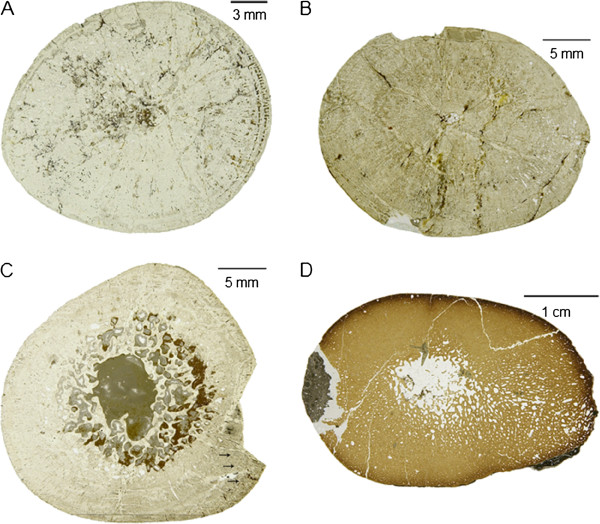
**Overview photographs of long bone thin sections in normal light of *Pistosaurus *and *Plesiosaurus*. A-D**, diaphyseal sections; **A-B**, *Pistosaurus* humeri (**A**, SIPB R 46, **B**, SMNS 84825); **C**, *Pistosaurus* femur (SIPB R 74); **D**, *Plesiosaurus* humerus SIPB R 90). In all images, ventral is at the bottom. The *Pistosaurus* humeri in A and B have a thick cortex and a very small medullary region unlike the *Pistosaurus* femur in C, which resembles the nothosaur femur in Figure 4C. The *Plesiosaurus* femur in D exhibits incipient osteoporosis, typical for pelagically adapted marine tetrapods.

**Figure 6:**
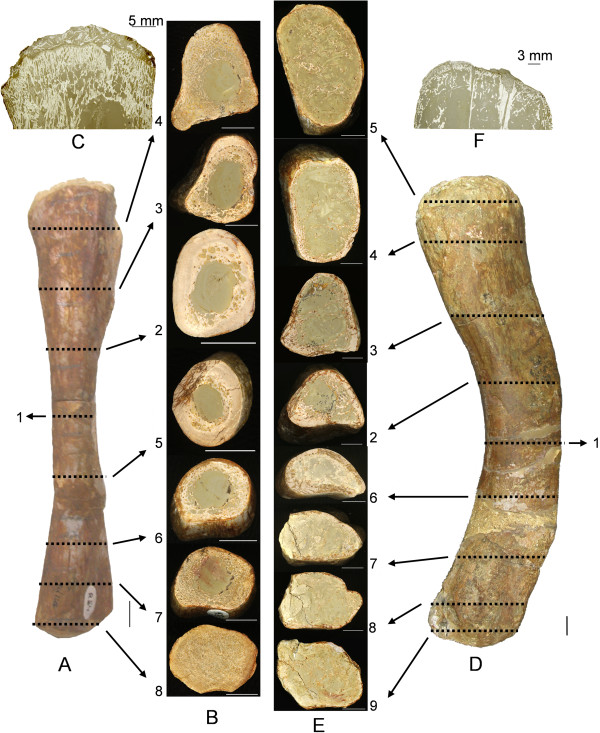
**Polished serial cross sections and epiphyseal longitudinal sections of *Nothosaurus mirabilis *long bones in comparison. A**, left femur (SIPB R 54/1) in dorsal view, anterior is to the right, proximal is to the top. **D**, left humerus (SIPB R 54/2) in dorsal view, anterior is to the right, proximal is to the top. Arrows in A and D assign the corresponding polished section to each section plane (marked by a dotted line). Serial transverse sections of femur (**B** 1–8) and of humerus (**E** 1–9) are numbered and described in the text from mid-diaphysis to proximal end and distal end, respectively. In all serial sections, anterior is to the right and dorsal side is up. Of B 1 and E 1 only mid-diaphyseal thin sections were produced which are pictured in Figure 3B and Figure 4B; **C**, longitudinal thin section of the proximal femur epiphysis. Plane of section is in anteroposterior direction. F, longitudinal thin section of the proximal humerus epiphysis. Plane of section is in dorsoventral direction. Femur sections B 1–8 display the generalized tetrapod long bone microanatomy. The cortex becomes thinner, and the amounts of secondary cancellous bone increase from the middle region of the shaft (**B** 1) towards both epiphyses (**B** 4 and B 8). The open medullar space enlarges from mid-diaphysis towards both articular ends and extends to the spongy endochondral and endosteal bone which provides the base for the articular cartilage (**C**). Humerus sections E 1–9 follow the same pattern as B 1–8, but throughout the entire humerus the cortex is much thinner, and secondary cancellous bone and endochodral and endosteal bone are only sparsely present. The medullary region extends proximally and distally to a very thin bony layer onto which the articular cartilage was placed in the living animal (**F**). Scale bars equal 10 mm.

Both, thin sections (Figure 3, 4, 5) and polished sections (Figure 6A-D) were produced to observe eosauropterygian bone microanatomy and histology, generally by removing a thin slice of bone with a rock saw. Before sampling the bones, their proximal and distal width and length, the shaft width and length, the total or preserved length, and shaft circumference were recorded (Additional file 1). Molds of the shaft regions were produced for all complete bones, so they could be restored after sampling by filling in the gaps with plaster. The samples were processed by standard petrographic techniques into thin sections. Thin sections were studied with a Leica DM EP® compound microscope in normal transmitted and polarized light. Photomicrographs of thin sections were taken with a Leica DFC420® digital camera mounted on the compound microscope and processed with Imagic Imageaccess®.

Additionally, serial polished sections of a *N*. *mirabilis* humerus (SIPB R 54/2) and femur (SIPB R 54/1) were produced. Their preparation and terminology is based on the work of Sander [43,70,71]. Polished sections were studied with incident light using a Wild M5A® binocular and overview images were produced by scanning them with a desktop scanner at 1200 dpi.

Because of the great range of the size of the medullary region observed in eosauropterygians, we devised a medullary index (MI, Table 1) to quantify medullary region size. Note that only the sections in the middle of the shaft were used, where the medullary region is the smallest. MI was calculated as the percentage of the dorsoventral diameter of the bone taken up by the dorsoventral diameter of the medullary region. MI was calculated from measurements taken from overview photographs of the thin sections. All other terminology used to describe bone microanatomy and histology follows Francillon-Vieillot et al. [64].

## Results

At the suprageneric level, the bone microanatomy and histology of the sampled sauropterygian taxa is described in the order of the phylogenetic hypothesis of Rieppel [4] and Liu et al. [5] (Figure 1B). Specimens of the different *Nothosaurus* species are described in order of decreasing stratigraphic age and increasing body size, since *Nothosaurus* intrarelationships are insufficiently resolved [16] (Figure 1C) to serve as an ordering criterion. The results are summarized in Table 1 and Additional file 1 which also includes detailed information about the samples.

### *Nothosaurus marchicus/Nothosaurus winterswijkensis* Humerus MfN R 174–2

MfN R 174–2 is a left humerus fragment of 44 mm length, lacking the distal end. The MI of this humerus is 42.5%, which indicates that the cortex is relatively thick (Figure 3A). The cortex consists of radially and longitudinally vascularized lamellar zonal bone tissue (RLLZB) and primary osteons are developed. The cortical bone shows seven growth cycles (Table 1). Beyond the third LAG, the bone tissue becomes markedly more organized. The amount of lamellar bone matrix increases, the vascularization decreases and growth marks show a closer spacing towards the bone surface.

**Figure 7:**
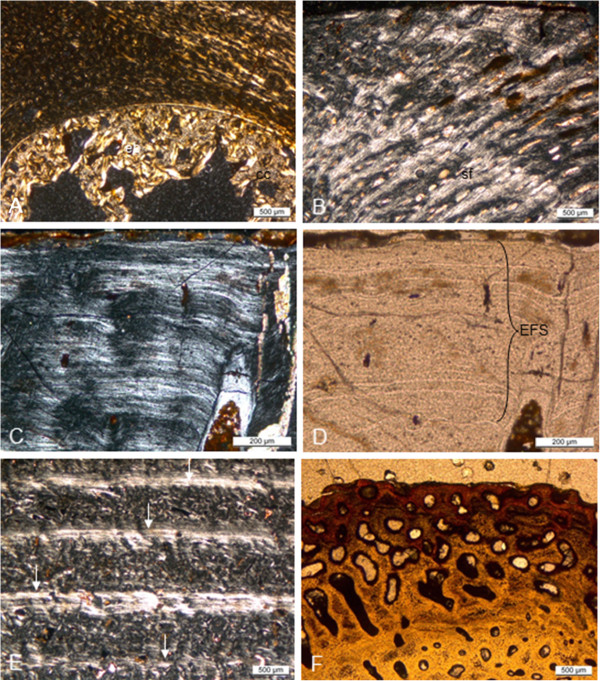
**Long bone histology of species of *Nothosaurus*. A-D**, thin sections in diaphysial region; **E, F**, thin sections in metaphyseal region; **A-C, E**, in polarized light; **D**, **F**, in normal light. **A**, humerus of *N*. *marchicus*/*N*. *winterswijkensi* (MfN R 174–2) showing a cortex which consists of well vascularized LZB. There is a thin, highly birefringent circumferential layer of lamellar bone that separates the medullary region from cortical bone. Trabeculae of endosteal bone with a core of calcified cartilage partially fill in the medullary region; **B**, humerus of *N*. *giganteus* (SIPB R 40), showing Sharpey’s fibres in cortical LZB of the posterior sector of the cross section. Note how the fibers influence the appositional bone organization, creating a radial appearance of the cortical bone, and how the vascular canals are deflected radially. Detail of humerus of *N*. *giganteus* (SIPB R 53) in polarized (**C**) and normal light (**D**). **C**, the outermost cortex of this specimen is composed almost entirely of thin layers of lamellar bone; **D**, closely spaced LAGs (EFS) in the outermost cortex of a humeral cross section indicate that diaphyseal growth has nearly completely ceased. **E**, humerus of *N*. *giganteus* (MHI 1903), Section 2 (the relatively more distal one), has a cortex composed of RLLZB showing regularly spaced zones, annuli and LAGs. Arrows mark the visible LAGs. **F**, humerus of *N*. *giganteus* (MHI 1903), Section 1 (the relatively more proximal one), the highly vascularized lamellar zonal cortex merges towards the shaft into cyclial LZB as seen in E. Abbreviations: cc, calcified cartilage, eb, endosteal bone, emb, embryonic bone, sf, Sharpey’s fibers.

**Figure 8:**
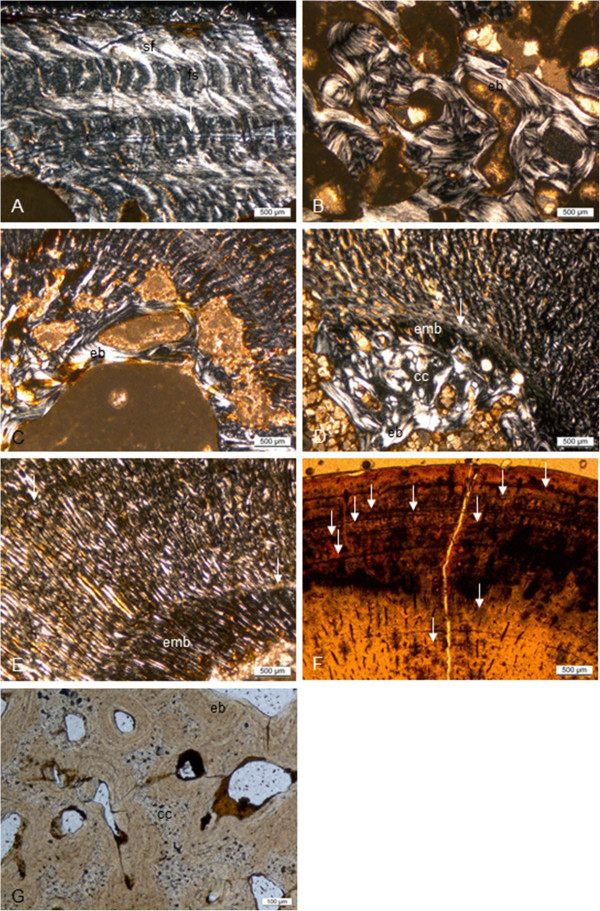
**Long bone histology of *Nothosaurus *species. A-G**, thin sections in diaphysial region; **A-E**, in polarized light; **F**, **G**, in normal light. **A**, humerus of *N*. *mirabilis* (SIPB R 54/2), cortex with radial vascular canals with funnel structures. Radial vascular canals undulate and seem to follow the orientation of the Sharpey’s fibers; **B**, medullary region of femur of *N*. *mirabilis* (SIPB R 54/2), showing secondary cancellous bone in the medullary region, consisting of several generations of endosteal lamellar bone; **C**, inner cortex and medullary region of femur of *N*. *mirabilis* (SIPB R 50/1), showing well vascularized LZB. Thick endosteal trabeculae line the medullary region. Large erosion lacunae are visible in deeper cortex; **D**, inner cortex and medullary region of humerus of *Nothosaurus* sp. indet. (MHI 1906). Embryonic bone is preserved up to the hatching or birth line (marked by the arrow). The remainder of the cortex is made up of highly vascularized LZB. Towards the center of the bone, a highly birefringent circumferential layer separates the cortical bone from the medullary region. It has partially been resorbed. In the medullary region, there are trabeculae consisting of endosteal bone and a core of calcified cartilage. **E**, humerus of *Nothosaurus* sp. indet. (MHI 633). Deposition of the cortex began with embryonic bone up to hatching or birth line (marked by the arrow). Cortical bone consists otherwise of highly vascularized incipient FLB; **F**, humerus of an adult individual of *Nothosaurus* sp. indet. (SMNS 84856). LAGs in the cortex (marked by arrows) become more closely spaced towards the bone surface, but there is no EFS in the outermost cortex; **G**, Endosteal lamellar bone surrounding cores of calcified cartilage in the medullary region of MfN R 174–2. Abbreviations: cc, calcified cartilage, eb, endosteal bone, emb, embryonic bone, fs, funnel structures, sf, Sharpey’s fibres.

The medullary region is separated from the cortex by a thin, highly birefringent layer of lamellar bone, which in some regions has subsequently been resorbed. Large amounts of calcified cartilage are preserved in the partially open medullary region. The calcified cartilage was slightly resorbed in parts and also partly encased by endosteal lamellar bone, forming trabeculae (Figure 7A, Figure 8G).

### *Nothosaurus mirabilis* humerus SIPB R 54/2

SIPB R 54/2 is a complete right humerus with the total length of 200 mm (Figure 6D), though part of the shaft was restored. We prepared a thin section of the mid-diaphysis in cross section, a longitudinal section of the proximal epiphysis in an anteroposterior plane, and nine serial cross sections to study microanatomical and histological variation along the bone.

(i) Microanatomy and histology of the mid-diaphysis

The MI of the humerus is 64%, indicating a rather thin cortex (Figure 3B), with the relatively thickest part situated posteroventrally. The cortex is composed of RLLZB. Some radial canals surrounded by “funnel structures” are scattered throughout the cortex (Figure 8A). Funnel structures is a descriptive term we use for parallel-fibered or lamellar bone surrounding vascular canals which appears sunken towards the center of the bone (see also [59]). Funnel structures result from the vascular canals exiting the bone in a pit. During apposition of the bone matrix, the pit grows outwards and results in a funnel shape. In parts, especially dorsally, radial vascular canals are more abundant than longitudinal canals. The primary osteons are immature to mature.

Ten growth cycles indicated by LAGs can be counted, but resorption has destroyed an unknown number of inner cycles (Table 1). The LAGs are spaced more closely beyond the sixth LAG.

The medullary region is lined with a thick layer of endosteal bone, especially on its dorsal and ventral sides. It is especially dorsally and posterodorsally filled with secondary cancellous bone, while it is mostly free anteriorly and ventrally. The secondary cancellous bone within the medullary region was formed by at least three resorption-reconstruction cycles recognizable by cross-cutting relationships (Figure 8B), but it also contains small fragments of primary cortex in the periphery. No calcified cartilage is preserved in the trabecular bone of the medullary region.

(ii) Microanatomy and histology of the proximal epiphysis

**Figure 9:**
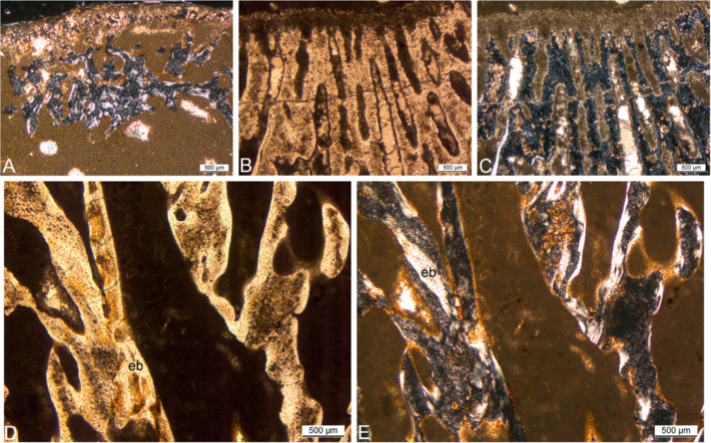
**Micrographs of longitudinal thin sections of *Nothosaurus mirabilis *long bones. A**, proximal epiphysis of *N*. *mirabilis* humerus (SIPB R 54/2) in polarized light. The section shows the growth plate below the articular cartilage. The endochondral bone is very thin at the proximal epiphysis, and there are only a few endochondral trabeculae, which have mostly been coated by endosteal bone. **B**-**E**, proximal epiphysis of *N*. *mirabilis* femur (SIPB R 54/1). **B** and **C** show the growth plate below the articular cartilage in normal transmitted and in polarized light. The trabeculae of the growth plate consist of cartilage covered by endochondral bone. **D**-**E**, trabeculae in deeper cortical regions of the femur in normal transmitted light (**D**) and polarized light (**E**). The trabeculae still have a calcified cartilage core but were subject to remodeling activity. Abbreviations: eb, endosteal bone.

In the proximal epiphysis (Figure 6D, F), the medullary region extends almost up to the proximal articular bone surface, and it is surrounded by rare, randomly deposited, mostly endosteo-endochodral spongiosa with little calcified cartilage, which has been modified by resorption and redeposition of lamellar bone. Below the articular surface, the spongiosa merges with a thin layer of very small and fine trabeculae consisting of calcified cartilage (Figure 9A). This spongiosa seems to be capped by a very thin layer of mostly lamellar bone, which was covered by the articular cartilage in the living animal. Large vascular canals perforate the proximal end of the humerus (Figure 6F) and formerly connected the articular cartilage with the medullary region [c.f. [37,72]].

(iii)  Microanatomy and histology observed in serial polished sections

The polished sections (Figure 6E, sections 1–9) reveal changes in microanatomy from the mid-diaphysis (section 1) to the proximal head of the humerus (sections 2 to 5) and then from the mid-diaphysis to the distal articular head (sections 6 to 9). Consistent with principles of long bone growth, the cortex greatly decreases in thickness both proximally and distally, while the medullary region expands. The secondary cancellous bone of the medullary region is most dense in the mid-shaft region with little cancellous bone in the metaphyses and a slight increase in the epiphyses.

### *Nothosaurus mirabilis* femora

Three *Nothosaurus mirabilis* femora were sampled: SIPB R 54/1 is a complete femur (165 mm in total length), but parts of the shaft were restored (Figure 6A). SIPB R 50/1 is a 120 mm long fragment of a left femur, consisting of the proximal head and a large part of the shaft. SIPB R 50/2 (120 mm long) is the distal head and part of the shaft of a right femur. SIPB R 50/2 is markedly larger than the two other specimens (Additional file 1). We prepared thin sections of the mid-diaphysis in cross section of all three specimens, a longitudinal section of the proximal epiphysis of SIPB R 54/1 in a dorsoventral plane, and eight serial cross sections of SIPB R 54/1 to study microanatomical and histological variation along the bone.

(i) Microanatomy and histology of the mid-diaphysis

The average MI of the femora is 46%, indicating a rather thick cortex (Figure 4B-C). The RLLZB of the cortex is well developed in all three femora. In SIPB R 54/1 and SIPB R 50/1, the inner cortex is more highly vascularized than the outer cortex. Primary osteons are developed to an intermediate degree. In the mid to outer region of the cortex, funnel structures are visible. Eight to nine growth cycles can be observed (Table 1) as well as several subcycles. The spatial arrangement of the LAGs is rather even, so there is no notable slowdown of growth, except possibly in SIPB R 54/1.

The open medullary region of SIPB R 50/1 is completely lined with a thin layer of two generations of endosteal lamellar bone (Figure 8C), despite the resorption cavities in the innermost cortex. In contrast, only about a third of the circumference of the medullary cavity of SIPB R 54/1 is lined with endosteal lamellar bone, the remainder having been destroyed by resorption. In SIPB R 50/2, the boundary between cortex and medullary region shows fresh resorption surfaces but no endosteal bone. A small, circular, open medullary cavity resides in the center of its medullary region. In the medullary region, secondary cancellous bone of endosteal origin is sparse to subordinate in both SIPB R 50/1 and SIPB R 54/1. In contrast, secondary cancellous bone, resulting from at least two resorption-reconstruction cycles, is prominent in SIPB R 50/2. SIPB R 50/1 and SIPB R 54/1 reveal a tendency towards osteosclerosis while the larger SIPB R 50/2 does not (Table 1).

(ii) Microanatomy and histology of the proximal epiphysis of SIPB R 54/1

The medullary region is positioned relatively anteroventrally in the proximal femoral head of *N*. *mirabilis* femur SIPB R 54/1 (Figure 6A, C), and it is surrounded by primary endochondral spongiosa. Towards the proximal articular surface, the spongiosa contains an increasing amount of calcified cartilage (compare with *Omphalosaurus*[37]) (Figure 9B, C). A thin layer of calcified cartilage, visible as very small and fine trabeculae, caps the proximal end of the epiphysis.

(iii) Microanatomy and histology of serial polished sections of SIPB R 54/1

The eight serial polished sections of the *N*. *mirabilis* femur SIPB R 54/1 (Figure 6A, B) reveal variation in cortex thickness and development of trabecular bone from the midshaft region towards the proximal and distal ends. The cortex of the mid-diaphyseal section (section 1, Figure 4B) is relatively thick, and only very sparse secondary cancellous bone is observable. The cortical bone in section 2 (Figure 6B) is similarly thick, but the cross section is larger, so the medullary region appears larger, too. Sparse secondary cancellous bone is present. Bone drift, probably due to formation and shifting of the trochanters, can be seen in section 3. Towards the proximal epiphysis, cortex thickness decreases, but the amount of secondary cancellous bone increases due to remodeling resulting from bone drift. In the most proximal section (section 4, Figure 6B), the cortical bone is thin, but secondary cancellous bone and the medullary region increase greatly. The same pattern is repeated towards the distal epiphysis of the femur.

### *Nothosaurus giganteus* humeri

Four *Nothosaurus giganteus* humeri (Additional file 1) were sampled: SIPB R 45, SIPB R 53, SIPB R 40, and MHI 1903. The first two were studied from cross sections of the mid-shaft region, but SIPB R 40 was sampled in the proximal region of the shaft. MHI 1903 was studied from two consecutive partial cross sections of the proximal metaphysis and epiphysis.

(i) Microanatomy and histology of the mid-diaphysis

The cortex of SIPB R 45, SIPB R 53, and SIPB R 40 is thin and composed of RLLZB (Figure 3C, D). Based on SIPB R 45 and SIPB R 53, the average MI of the *N*. *giganteus* humeri is 68% (Table 1). The MI of SIPB R 40 could not be reliably measured because of the proximal location of the section. The cortical bone of SIPB R 45 is more highly vascularized than that of SIPB R 40, which is in turn more highly vascularized than that of SIPB R 53. Primary osteons in SIPB R 40 are immature but mature in SIPB R 53, and funnel structures can be observed throughout the whole cortex, as in the *N*. *mirabilis* humerus (Figure 8A). The cortical bone generally becomes more highly organized towards the bone surface (Figure 7C). Both SIPB R 53 and SIPB R 45 show an external fundamental system (EFS) (Figure 7C, D, Table 1).

The medullary regions are open, although sometimes sparse secondary trabeculae of at least two resorption-reconstruction cycles was deposited randomly within the medullary cavity (Figure 3C, D). No calcified cartilage is preserved in the trabecular bone. The medullary region of SIPB R 45 is partly lined by a very thin layer of endosteal lamellar bone. In contrast, SIPB R 40 shows a thick, but discontinuous layer of circumferential endosteal bone only in some places, and SIPB R 53 shows none.

(ii) Microanatomy and histology of metaphysis and epiphysis

The proximal head and the metaphysis of humerus MHI 1903 was cut into two consecutive cross sections. Section 1 (Figure 3E) is positioned more proximal than section 2. The cortex is thin in both sections and consists of LZB. In section 1, highly vascularized zones alternate with less vascularized, thinner and more highly organized annuli, in each of which a LAG is found. The LAGs are relatively evenly spaced, indicating cyclical growth at a rather constant rate (Figure 7E). Vascular canals have sometimes a reticular orientation but more often a longitudinal one. This is similar to section 2, but its vascular canals are smaller and mainly oriented longitudinally and seldom reticular.

Towards the anterior side of section 1, the LZB grades into primary coarse cancellous bone which also shows the growth cycles that are seen in the LZB (Figure 7F). Resorption cavities are observable, especially anteriorly in the deeper cortex.

### *Nothosaurus giganteus* femur IPB R 49

SIPB R 49 is a complete right femur. At a total length of 230 mm, it is the largest femur sampled (Additional file 1). The MI is 76% which indicates a similarly thin cortex as described for the humeri of the same species (Figure 4A). The cortex, which consists of RLLZB, is thickest on the dorsal side, and it is poorly vascularized. The outer cortex shows mainly mature longitudinal primary osteons. LAGs are relatively evenly spaced, indicating rather constant growth rates. Seven growth cycles can be counted (Table 1), some of which show subcycles.

The medullary region contains much secondary cancellous bone with a very small open medullary cavity in the center. The bone trabeculae were formed by at least two resorption-reconstruction cycles (Figure 8B). No calcified cartilage is preserved in the trabecular bone of the medullary region.

### Small *Nothosaurus* sp. indet. humerus MHI 1906

MHI 1906 is the distal half of a small left nothosaur humerus (Additional file 1). The MI of the specimen is 35%, but because most of the original bone surface was not preserved, the MI originally must have been lower (Figure 3F). The cortical bone can be differentiated into two regions. Up to the first growth mark, the bone tissue probably consists of embryonic bone. In polarized light, this tissue stays opaque and can be identified as woven-fibered bone. We interpret the first growth mark as the neonatal line, recording hatching or birth (compare with [65,68,73]). Note that we do not know whether nothosaurs were oviparous or viviparous, with their sistergroup, the pachypleurosaurs, having been life-bearing [74]. Beyond the neonatal line, the cortex is made up of RLLZB (Figure 8D). The primary osteons are very immature. Bone tissue organization increases towards the bone surface, and three growth cycles are observable (Table 1). The medullary region is only partially open and surrounded by a thin lining of endosteal bone, which is only preserved on the ventral side. Posteriorly and dorsally, it had been resorbed, and endosteal bone was deposited instead. Within the medullary region, a small amount of calcified cartilage is preserved, which was slightly resorbed in parts and partially lined with secondary endosteal bone of two generations (Figure 8D, and compare to Figure 8G). This humerus thus fits the definition of osteosclerosis (Table 1). Its histology suggests that the humerus pertains to a juvenile of a larger species.

### Small *Nothosaurus* sp. indet. humerus MHI 633

MHI 633 is the distal half of a small left nothosaur humerus (Additional file 1). The MI of this specimen is very low at 27%, so the cortical bone is thick (Figure 3G). The cortex consists of incipient fibrolamellar bone tissue (FLB), unlike all the other sampled *Nothosaurus* long bones. The first growth mark, in the inner region of the cortex, probably is the neonatal line. The probably embryonic bone consists of woven-fibered bone tissue (Figure 8E). Beyond the neonatal line, there is a woven-fibered bone matrix surrounding immature primary osteons (Figure 8E), hence the notion of “incipient FLB”. Three growth cycles could be counted (Table 1). There is no outward increase in bone tissue organization. Based on Amprino’s Rule (e.g., [75,76]), this bone grew at a relatively high and rather constant rate. The cortex is well vascularized with radial canals, much more so than in the other *Nothosaurus* long bones. Cell lacunae in the incipient FLB are rounded and plump and show no preferred orientation. The radial vascular canals pierce the bone surface, creating a pitted surface.

The cortex is set off abruptly and irregularly from an open medullary cavity (compare to [77], plate 1, Figure 3). The inner margin of the cortex lacks any evidence of resorption, and there is no endosteal bone lining the medullary region. The thin cementing line between medullary region and cortex, as seen in MHI 1906 and MfN R 174–2, cannot be observed. No calcified cartilage is preserved. This microanatomy and histology is unique among the sampled *Nothosaurus* long bones and is most consistent with an early juvenile stage of the specimen, before perimedullary resorption and calcification of cartilage set in. The open medullary cavity may have been filled with uncalcified cartilage that was completely removed during decay and fossilization.

Because humerus MHI 633 experienced no perimedullary resorption activity, it would seem to follow the pathway to osteosclerosis [29,30,34]. However, we refrain from applying this term to the specimen because of its very immature nature and the strong vascularization. Without quantification of bone compactness and porosity (which is beyond the scope of this study), it is uncertain whether MHI 633 does indeed shows bone mass increase relative to the terrestrial condition or whether the strong vascularization compensates for the apparent gain in bone mass caused by the absence of perimedullary resorption activity.

### Small *Nothosaurus* sp. indet. femur SMNS 84856

SMNS 84856 is an incomplete right femur (Additional file 1). The MI of the specimen is 28%, indicating a thick cortex (Figure 4D) which is composed of RLLZB. Primary osteons are developed to an intermediate degree, and funnel structures are present (Figure 8A). In the outer periphery of the cross section, ten growth cycles can be counted (Table 1). The spacing of the LAGs (Figure 8F) becomes narrower towards the outer bone surface, and the organization of the bone tissue increases (Table 1). The medullary region is almost completely filled with secondary endosteal bone of two generations. SMNS 84856 thus exhibits osteosclerosis (Table 1).

### *Pistosaurus longaevus* humeri

**Figure 10:**
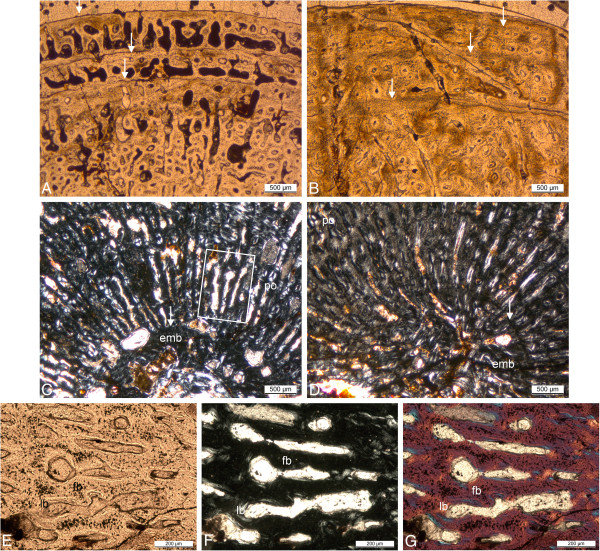
**Comparison of the humerus histology of two *Pistosaurus longaevus *individuals.** Match the juvenile SIPB R 46 (**A**, **C**) and the adult SMNS 84825 (**B**, **D**). **A**-**B**, outer cortex in normal transmitted light. Radially and longitudinally vascularized FLB is interrupted by several thick parallel-fibred annuli (marked by arrows). Vascular spaces are filled in by more highly organized bone tissue to a greater degree in the older individual (**B**) than in the juvenile (**A**). **C**, **D**, inner cortex in polarized light. Embryonic bone is preserved in both individuals and extends to the hatching or birth line (marked by arrow). The rest of the cortex consists of strongly vascularized FLB with distinctly radial primary osteons. Although the medullary region is lined by circumferential layers of endosteal bone in both specimens, the cortex of the juvenile (**C**) is more highly vascularized than that of the older one (**D**). **E**-**G**, detail (box in **C**) of the FLB of the juvenile (SIPB R 46) showing the framework of woven-fibered bone, which is only incompletely filled in by lamellar bone. The primary osteons are thus still incompletely formed. The same view is seen in normal-transmitted light in E, in polarized light in F, and with the gypsum plate in G. Abbreviations: emb, embryonic bone, fb, fibrous or woven-fibered bone, lb, lamellar bone, po, primary osteon.

One complete (SIPB R 46) and one incomplete humerus (SMNS 84825) of *Pistosaurus* were sampled, with the former representing a smaller individual than the latter (Additional file 1). The MI is extremely low at around 6% (Figure 5A-B), indicating a very small medullary region. The very thick cortex is composed of a woven bone matrix with radial vascular canals that are filled in by centripetal deposition of lamellar bone (Figure 10A-G). These structures thus represent primary osteons, and the tissue is FLB. The FLB is cyclically interrupted by growth marks. While the cortical bone of *Pistosaurus* is quite comparable to that of dinosaurs and large mammals [e.g., [41,64]], it differs in that the primary osteons are mainly radially oriented. *Pistosaurus* humeri are markedly higher vascularized than nothosaur long bones. Primary osteons of SIPB R 46 (Figure 10A, C) are rather immature in comparison to SMNS 84825 (Figure 10B, D). Secondary osteons are absent in *Pistosaurus* humeri.

Because of the very small medullary region, it is clear that the first growth mark (Figure 10C, D) must represent the neonatal line in both specimens. The embryonic bone is moderately vascularized woven-fibered bone. In both specimens, growth marks consisting of an annulus with an indistinct LAG are spaced closer together with increasing body size, and finally, in the outermost periphery of the cortex, thick avascular parallel-fibered annuli alternate with zones of FLB with longitudinal and very short radial vascular canals (Figure 10A, B). This indicates that diaphyseal growth slowed down in the fourth to fifth growth cycle. Five growth cycles (Table 1) were counted in the small specimen and seven in the larger specimen.

The medullary region is open and lined by endosteal lamellar bone in SMNS 84825, but in SIPB R 46 it is filled and contains very sparse remnants of calcified cartilage incorporated into endosteal bone. The very small medullary cavity of the *Pistosaurus* humeri obviously results from a suppressed perimedullary resorption activity, which is one of the conditions leading to osteosclerosis. However, as in the case of MHI 633, and based on the same arguments, we refrain from employing the term osteosclerosis in the case of *Pistosaurus*. This is because the decrease in resorption activity may have been compensated for by the increase in vascularity in the FLB of the cortex.

### *Pistosaurus longaevus* femur SIPB R 74

**Figure 11:**
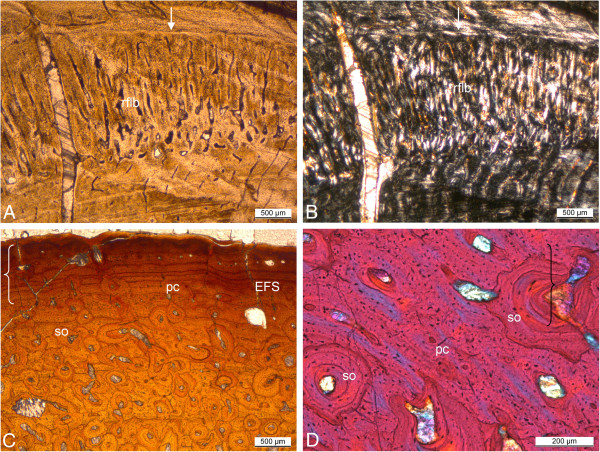
**Bone histology of *Pistosaurus longaevus *and *Plesiosaurus dolichodeirus*. A** and **B**, *Pistosaurus longaevus* femur (SIPB R 74), seen in normal-transmitted (**A**) and polarized light (**B**). **C** and **D**, *Plesiosaurus dolichodeirus* femur (SIPB R 90) seen in normal-transmitted light (**C**) and with gypsum plate (**D**). **A** and **B**, detail of the lower right sector in cross section in Figure 5C. Unlike the humeri, the cortex of the femur consists of LZB except for the deepest cortical regions. The detail shows a growth cycle that locally consists of radial FLB following one of LZB. **C**, cortical bone composed of FLB of *Plesiosaurus dolichodeirus* (SIPB R 90). The primary cortex was subject to Haversian remodeling, partially obscuring it. An EFS is observable. Note the relatively small primary vascular canals and the large secondary osteons. **D**, detail of SIPB R 90, showing the radial fabric of primary bone, which appears to have been inducing resorption activity that led to the formation of secondary osteons. Abbreviations: pc, primary vascular canal, rflb, radial fibrolamellar bone, so, secondary osteon.

SIPB R 74 is an incomplete *Pistosaurus* femur (Additional file 1). At 66%, the MI of the femur is much higher than that of the humeri of *Pistosaurus* (Figure 5C). The femoral cortex is mostly made up of poorly vascularized RLLZB (Figure 11A, B) instead of the FLB of the humeri. Only in the deep regions of the femoral cortex, sparse woven-fibered bone matrix can be observed. Superficially, the cortical bone shows mainly funnel structures. In the deeper region, mature longitudinally oriented primary osteons are found. Eight growth cycles (Table 1) and several subcycles can be observed. The LAGs in the LZB part of the cortex are relatively evenly spaced. The increase in bone tissue organization, from FLB to LZB, records a slowdown in growth. In the center of the medullary region, there is a small medullary cavity surrounded by secondary cancellous bone of at least two generations of endosteal lamellar bone and of interstitial primary cortex.

One side of the cross section (we do not know which, because we are unable to orient the *Pistosaurus* femora in dorsoventral direction, see above), records a peculiar growth spurt in the outer cortex (Figure 11A, B). Radially vascularized FLB is partially filled in with lamellar bone matrix. The deposit shows four subcycles. Each of them ends in a thin layer of lamellar bone. Laterally in the section, the first three subcycles merge into the seventh growth cycle and the fourth subcycle and the eighth growth cycle converge. In the eighth growth cycle, the individual had died.

Similar observations of radially vascularized FLB in the outer cortex and recording local growth spurts have also been reported by Erickson and Tumanova [78] and Klein and Sander [71] for *Psittacosaurus* and *Plateosaurus*, respectively. In the *Pistosaurus* femur, this bone tissue is deposited onto LZB, whereas in *Psittacosaurus* and *Plateosaurus*, it is deposited onto FLB with laminar vascularization. Growth of radially vascularized FLB is associated with a rapid change of morphology, possibly due to a fast ontogenetic relocation of muscle insertion areas [71,78]. The presence of some Sharpey’s fibers in radially vascularized FLB bone of the *Pistosaurus* femur is consistent with the muscle insertion hypothesis.

### *Plesiosaurus dolichodeirus* femur IPB R 90

IPB R 90 is a plesiosaur femur 150 mm long (Additional file 1). The MI is about 35% (Figure 5D). However, the cross section appears relatively spongy (typical for secondarily marine tetrapods (compare with, e.g., [38,64]), and the transition from the medullary region to the cortex is very gradual (Figure 5D). Unlike in all previously discussed specimens, the cortex is subject to intensive Haversian remodeling, with only limited areas of primary cortex remaining. These are located beneath the bone surface and interstitially between the secondary osteons. The primary cortex consists of well vascularized FLB. The primary osteons show a preferred radial orientation, but are not strictly radial (Figure 11C). In this way they differ from the radial vascularization in *Pistosaurus* and *Nothosaurus*. The primary osteons are unusual and differ from those of *Pistosaurus* and typical FLB in that the centripetal infill of the vascular canals does not only consist of lamellar bone but also of parallel-fibered bone (Figure 11D). The outer periphery of the cortex shows closely spaced growth marks forming an EFS (Figure 11C).

Locally, there are at least three generations of secondary osteons as indicated by cross-cutting relationships. The cementing lines that separate the osteons from the primary cortical bone and from each other are undulating and irregular (Figure 11C, D). Although most secondary osteons are oriented longitudinally, resorption activity leading to the first generation of secondary osteons appears to have been controlled by the radial fabric of the primary bone. This leads to an arrangement of the secondary osteons in radial rows (Figure 11D). Towards the anterior and posterior side, secondary osteons appear elliptical, indicating an oblique orientation. Their longer axes point towards the medullary region and towards either the anterior or the posterior side. This leads to the overall impression that the cortex is rather radially vascularized. In the medullary region, there is secondary cancellous bone produced by intensive remodeling. The *Plesiosaurus* femur appears to be osteoporotic in some regions of the cortex but not in all. Osteoporosis is not as prevalent as shown by e.g., Wiffen et al. [38] and Buffrénil and Mazin [37] for other plesiosaurs but the more remodeled the cortex is, the more vascularized it becomes.

### Quantitative comparison of medullary region size in Eosauropterygia

**Figure 12:**
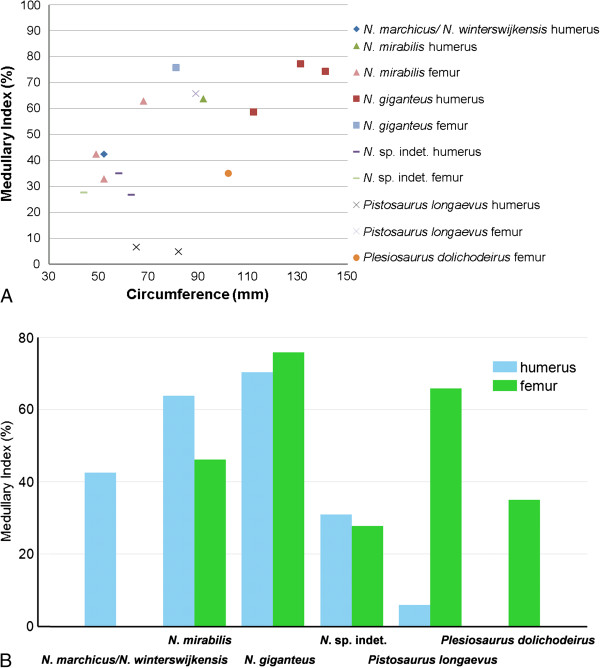
**Variation of medullary index. A**, Scatter plot of medullary index (MI) vs. bone circumference of all sampled sauropterygian long bones, subdivided by genus, species and long bone type. Three clusters can be distinguished, reflecting either very small, intermediate, or large medullary regions. Note the trend of increasing MI with increasing body size in the genus *Nothosaurus*. *Pistosaurus* differs from the other sauropterygians in its very small medullary region in the humerus. **B**, comparison of the MI for each bone type of each species. The highest MI is seen in the long bones of *Nothosaurus giganteus*. Also note the great differences between the MI of the humeri and the femur of *Pistosaurus*.

As noted, long bones of *Nothosaurus*, *Pistosaurus,* and *Plesiosaurus* show great variation in relative size of their medullary region. In particular, two Upper Muschelkalk nothosaur species of large body size, *N*. *giganteus* and *N*. *mirabilis*, greatly decrease cortical thickness by increasing medullary space especially in their humeri. Three clusters of relative medullary region size can be differentiated based on MI (Figure 12A). The first represents the humeri of *Pistosaurus* with their extremly low MI (less than 10%). The second group consists of the long bones of *Nothosaurus* sp. indet., the two smaller femora of *N*. *mirabilis* (SIPB R 50/1 and SIPB R 54/1), the *N*. *marchicus*/*N*. *winterswijkensis* humerus, and the Lower Jurassic *Plesiosaurus dolichodeirus* femur, all of which have intermediate MIs ranging from approximately 25% and 45% (Figure 12A). The third group comprises all long bones of *N*. *giganteus*, the humerus and the large femur of *N*. *mirabilis* (SIPB R 50/2), and the femur of *Pistosaurus*, which have very high MIs ranging from 58% to 77% (Figure 12A). A plot of MI against minimal shaft circumference (MSC), as a proxy of body size (Figure 12A), shows a week trend of increasing MI with increasing MSC.

## Discussion

### Biomechanical implications of *Nothosaurus* microanantomy

Our histological study of *Nothosaurus* long bones shows that biomechanical adaptations evolved within the genus during the Middle Triassic and that the changes are correlated with increasing body size [79]. The plesiomorphic state is seen in *N*. *marchicus*/*N*. *winterswijkensis* (MfN R 174–2) from the Anisian (Lower Muschelkalk) as well as the small femora (SIPB R 54/1 and SIPB R 50/1) of *N*. *mirabilis* and the humerus and femur of *Nothosaurus* sp. indet. (MHI 1906 and SMNS 84856) from the Upper Muschelkalk (Ladinian). These bones show lamellar zonal bone tissue with mainly longitudinal vascular canals and differing degrees of cortical thickening (Figure 12) and bone mass increase (i.e., limited endosteal resorption, incomplete endochondral ossification resulting in calcified cartilage, or dense secondary tissue in the medullary region) (Table 1). Character polarity is known from work on pachypleurosaur bone histology [53,59,60].

The derived state is represented by the humeri of large-bodied Upper Muschelkalk species of Ladinian age, namely *Nothosaurus giganteus* and *N*. *mirabilis* and the femur of *N*. *giganteus*. These share a very thin cortex and an unusually large medullary cavity (MI approx. 60 to 80%, Figure 12) with few trabeculae, resulting in greatly decreased skeletal density. This suggests that *Nothosaurus* evolution saw a shift in lifestyle and habitat. While the small-bodied basal nothosaur species from the Lower Muschelkalk adapated to shallow water by an increase in bone mass, the large-bodied and derived species from the Upper Muschelkalk moved into the open marine environment and became more active swimmers, for which bone mass decrease was adaptive. The small-bodied species from the Upper Muschelkalk retained the ancestral condition of living in shallow water. Among the large-bodied Upper Muschelkalk species these evolutionary changes seem to have been recapitulated in their ontogeny (see below).

The histological specialization of achieving low skeletal density by extreme resorption of the cortex, described here for the two large-bodied Upper Muschelkalk *Nothosaurus* species, is unique among secondarily aquatic tetrapods and has not been described before. While low density of skeletal elements evolved convergently several times in secondarily aquatic tetrapods and represents the terminal adaptation to the aquatic environment [e.g., 28, 29, 30, 34], in all the other instances bone mass decrease evolved by the transformation of compact bone to cancellous as seen in, e.g., extant whales [28] and ichthyosaurs [34]. However, their cortex consists of FLB instead of LZB. Since there is no recent analog for the large-bodied *Nothosaurus* microanatomy, it is unclear what kind of soft tissue filled their large medullary cavity. A likely candidate is fatty yellow bone marrow which would have decreased the density of these bones compared to a fill of red bone marrow in the juveniles.

Bone morphology and microanatomy are fundamentally influenced by mechanical factors. According to Wolff’s law, bone will be resorbed if loading decreases [80]. Thus, if bone density and the amount of bone tissue remain constant but the diaphyseal diameter increases, the bone wall becomes thinner and at the same time the bone will become adapted to resist higher torsional loads (e.g., [81]). The large, triangular cross section of *Nothosaurus* humeri (not seen in any other sauropterygians) and the shape of the bone wall provided a mechanically strong construction despite the thinness of the cortex. This peculiar bone microanatomical specialization would have been able to withstand high mechanical loads despite low skeletal mass.

This suggests that the forelimb played an important role in the locomotion of *N*. *giganteus* and *N*. *mirabilis*[79], a view that is also supported by the complex morphology of the nothosaur humerus [22,62] and nothosaur muscle reconstructions [25,26]. We hypothesize that this adaptation to high bending loads indicates either underwater flight, as employed by sea turtles [82] and penguins [83,84], or the so-called “rowing flight” employed by sea lions [27]. In addition, the generally lower density of skeletal elements in the two large-bodied Upper Muschelkalk *Nothosaurus* species suggests that these nothosaurs were able to swim faster and more energy-efficient than the smaller *Nothosaurus* species.

The distinct differences in bone microanatomy and histology of long bone shafts between large-bodied *Nothosaurus* and similar-sized Plesiosauria indicate that lightening of the skeleton evolved convergently in the two lineages. The pressing question though is, why lightening of the skeleton in *Nothosaurus* evolved along such a different pathway compared to all the other secondarily aquatic and pelagic tetrapod clades i.e., some adult Plesiosauria, Ichthyosauria, Mosasauridae, some Chelonioidea, and Cetacea. These groups all convergently evolved the same solution, highly cancellous bone and a reduction of the cortex, but at different times in geologic history [34]. Possibly, the evolutionary pathway to completely cancellous bone is linked to a high basal metabolic rate and endothermy, which obviously preceeded the aquatic adaptation in cetaceans, but may have evolved concurrently in ichthyosaurs, plesiosaurs and possibly in mosasaurs [85]. Today’s Chelonioidea with the exception of *Dermochelys* seem to pose an exception to this hypothesis because they are ectothermic but have spongy long bone shafts. Bone microstructure in *Dermochelys* is consistent with our hypothesis because of its increased basal metabolic rate, which is three times that of a typical reptile [86,87].

### Ontogenetic implications of *Nothosaurus* histology and microanatomy

(i) Assigning individuals to biological ontogenetic stages

The questions arise whether the observed microanatomical and histological differences between individuals and taxa may be due to different ontogenetic stages and whether these differences can be used to determine the biological ontogenetic stage of eosauropterygian specimens. We suggest that counting growth marks (Table 1) and describing and interpreting bone tissue types (Table 1) do indeed permit identifying the individuals as juvenile, subadult, adult, and fully grown.

Although we do not know what the growth curves of either *Nothosaurus*, *Pistosaurus,* or *Plesiosaurus* look like, it seems to be reasonable to presume that they followed the general plesiomorphic reptilian pattern of growth, including the timing of sexual maturity (see discussion in the Background section). With the onset of sexual maturity, resource allocation shifts from gaining size to reproduction, which in turn is reflected by the closer spacing of growth marks [40,42]. Accordingly, we identified individuals with very few LAGs and highly vascularized bone tissue as juveniles. Individuals with several regularly spaced LAGs, decrease in vascularization, and increase in bone tissue organization were interpreted as subadults, approaching sexual maturity. Individuals that record a sudden decrease in growth rate, e.g., by a sudden decrease in LAG spacing, were interpreted as adults (though not fully grown). Finally, individuals that show a cessation of growth (EFS) were termed fully grown. There were no obviously senescent individuals in this study.

(ii) The qualitative and quantitative record of growth in *Nothosaurus* long bones

The two small humeri MHI 633 and MHI 1906 come from juvenile individuals because only two LAGs (neonatal line + one LAG) are present. Since the two specimens are both of roughly the same size, but also of roughly the same stratigraphic age and coeval with the larger-bodied *Nothosaurus* species*,* we hypothesized that the earlier juvenile (MHI 633) belongs to the very large *N*. *giganteus* and the later juvenile (MHI 1906) belongs to the large *N*. *mirabilis*. According to the criteria introduced above, the femora SIPB R 49, SIPB R 50/1, and SIPB R 50/2 had not reached sexual maturity yet and are therefore identified as subadults. The humeri MfN R 174–2 (from the Lower Muschelkalk), SIPB R 40, and SIPB R 54/2, the femur SMNS 84856 and possibly also the femur SIPB R 54/1 record a slowdown in growth rate, suggesting sexual maturity [53,65,68]. Hence, the specimens were assigned to the “adult” category. Notably, SMNS 84856 is the smallest femur in the sample but on the basis of its bone histology also one of the most mature. The humeri SIPB R 45 and SIPB R 53 are interpreted as fully-grown specimens because bone growth has almost ceased as recorded by the deposition of an EFS.

The highest number of LAGs (10) was counted in SIPB R 54/2 and SMNS 84856. However, because the former shows perimedullary resorption and neither of them shows an EFS, we estimate a time frame of 15 to 20 years for the larger nothosaur species (*N*. *mirabilis*, *N*. *giganteus*, *Nothosaurus* sp. indet.) before growth stopped, taking several resorbed growth marks and several growth marks yet to be deposited into account.

(iii) The juvenile *Nothosaurus* humeri and their evolutionary implications

The hypothesis that MHI 633 and MHI 1906 are juveniles of two species of *Nothosaurus* that differ in adult size, i.e., *N*. *mirabilis* and *N*. *giganteus*, has implications for body size evolution in *Nothosaurus* via heterochrony. The cortex of the juvenile *N*. *mirabilis* (MHI 1906) is composed of LZB and that of the juvenile *N*. *giganteus* (MHI 633) of FLB. Thus MHI 633 grew faster than MHI 1906 in the same amount of time, because general bone apposition rate is higher for FLB than for LZB. The large body size of *N*. *giganteus* evolved due to an acceleration of growth during early ontogeny (MHI 633, FLB), allowing *N*. *giganteus* to attain a larger body size than basal nothosaurs from the Lower Muschelkalk, but in the same time. After the juvenile stage, *N*. *giganteus* growth slowed down and LZB was laid down.

The relatively large body size of *N*. *mirabilis* may also have been reached by acceleration. Compared to a more basal Lower Muschelkalk *Nothosaurus* species such as *N*. *marchicus*/*N*. *winterswijkensis*, *N*. *mirabilis* may have grown faster in its first few years, laying down the highly vascularized LZB with a high amount of parallel-fibered bone matrix seen in MHI 1906.

The juvenile *N*. *mirabilis* and *N*. *giganteus* specimens have a comparably thick cortex (MI 25-35%) while the large humeri assigned to these two species have a thin cortex (MI of 58% and 77%). This suggests that there was an ontogenetic shift in lifestyle and habitat in these two species. While the juveniles may have lived closer to shore and were bottom-dwelling, the adults shifted to a more pelagic lifestyle in the water column. Such a shift has previously been suggested by Wiffen et al. [38] for plesiosaurs from the Upper Cretaceous of New Zealand and by Katsura [88] for champsosaurs from the Late Cretaceous of Montana, USA.

### Ontogenetic implications of *Pistosaurus* and *Plesiosaurus* histology

Histology suggests that no juvenile individuals of these two taxa were sampled. In the *Pistosaurus* femur SIPB R 74, the eight growth marks are spaced evenly, and no sudden decrease of vascularization can be recognized. However, there is an outward change in bone tissue from FLB to LZB, so the individual was subadult or adult, but not fully grown. The uncertainty stems from the fact that the specimen is not part of a growth series. The humeri SMNS 84825 and SIPB R 46 must have belonged to adult individuals because they had reached sexual maturity as suggested by the slow-down in growth in the fourth to fifth cycle. In terms of age at death, the *Pistosaurus* samples suggest similar ages as those recorded by the *Nothosaurus mirabilis* samples.

The EFS in the femur of *Plesiosaurus* indicates that the individual was fully grown. At least ten growth cycles can be counted, including those of the EFS, but most of the earlier growth mark record was destroyed by intense cortical remodeling.

As in *Nothosaurus*, the humerus of *Pistosaurus* shows a more derived histology (FLB over LZB) than the femur. However, the humerus exhibits reduced perimedullary resorption activity while the femur reveals a rather similar MI to the larger of the two *N*. *mirabilis* femora (SIPB R 50/2). These differences suggest that humerus and femur also differed in their function. It also underscores the notion that postcranial skeletal adaptations to a secondarily aquatic lifestyle first happened in the forelimb of stem-group sauropterygians, particularly in the humeri, and were linked to the evolution of different modes of locomotion [22-26] and/or sexual dimorphism and different morphotypes [33,59,62,74,89].

### Fibrolamellar bone in *Pistosaurus* and the evolution of the Plesiosauria

The most surprising result of this study was the fundamental differences between the microanatomy and histology of *Pistosaurus* long bones, particularly the humerus, and those of *Nothosaurus* (as already briefly announced by [79]). The highly vascularized and nearly continuously deposited FLB of the *Pistosaurus* humerus suggests high growth rates. FLB also suggests endothermy (e.g., [36,52,77,90,91]) because fast growth requires a high basal metabolic rate. This implies that the high basal metabolic rate of plesiosaurs [85] arose in the last common ancestor of Plesiosauria and Pistosauridae, although an earlier origin among basal Pistosauroidea is possible [59].

We also found abundant FLB in *Plesiosaurus dolichodeirus*. Unlike in *Pistosaurus*, the primary cortex of *Plesiosaurus* was subject to extensive Haversian remodeling. A bone tissue consisting of woven-fibered bone but apparently lacking primary osteons was described in plesiosaurs from the Upper Cretaceous [38]. These specimens also show intense Haversian remodeling. Thus, our *Plesiosaurus* sample moves the record of fast growing woven-fibered bone tissue (as a component of FLB) in the plesiosaur lineage back to at least the Lower Jurassic. Our *Pistosaurus* samples show that this tissue had already evolved in sauropterygians by the Middle Triassic, at least 30 million years before the plesiosaur radiation. In fact, some Lower Muschelkalk (Anisian) sauropterygian long bones that may belong to the basal pistosauroid *Cymatosaurus* or a closely related taxon also show some FLB, albeit in a zonal pattern alternating with LZB [59].

To understand the origin of FLB and high growth rates in Eosauropterygia, their sistergroup, the Placodontia needs to be considered. As for plesiosaurs, placodont bone histology remains little studied, although there is evidence that placodonts also had fast-growing bone tissue [59,92]. Thus, at least intermittent deposition of FLB is present in at least three different lineages of early Sauropterygia (Placodontia, Pistosauroidea and in the pachypleurosaur *Anarosaurus heterodontus*) [59]. Our observation of uninterrupted FLB in a juvenile of a large-bodied nothosaur, presumably *N*. *giganteus*, indicates that all sauropterygians were able to lay down this bone tissue at least during early stages of their life history, but only in the Pistosauria, sustained deposition of FLB appears to have evolved.

Until recently, *Pistosaurus* was the only representative of the Pistosauridae, and its occurrence was restricted to rare finds from the Upper Muschelkalk beds (Middle Triassic). However, finds from the Middle Triassic of Nevada (*Augustasaurus*; [12,18]) and of China [11] indicate that the Pistosauridae had already spread over the entire northern hemisphere by the Middle Triassic. This suggests the ability of pelagic dispersal, which may have included a certain cold-water tolerance [19,59]. Nothosauroidea, in contrast, remained restricted to the warm waters of the Tethys and surrounding epicontinental seas throughout their evolutionary history and went extinct at the end of the Middle Triassic.

Recently, Klein [59] hypothesized that it was the evolution of a high basal metabolic rate in the most basal Pistosauria such as *Cymatosaurus* and *Anarosaurus* that permitted the plesiosaur lineage to achieve a pelagic lifestyle already during the Middle Triassic and to survive into the Jurassic. However, here we refine this hypothesis by noting that another prerequisite for a pelagic lifestyle is a substantial increase in body size from the generally small (<1 m total length) basal pistosauroids to the larger Pistosauria (> 2 m total length). This is based on the observation that all pelagic marine mammals of today are in the size range of Pistosauridae and Plesiosauria and that there are no open-ocean marine mammals the size of *Cymatosaurus*, let alone *Anarosaurus*. The ability to grow quickly as recorded by FLB may have been the prerequsite to large body size.

Together, large body size, high growth rates and presumably high basal metabolic rates having been present in Pistosauridae suggest that at least some Plesiosauria also possessed these features, which is indeed the case [38]. We hypothesize that these features were the key to the evolutionary success of the Plesiosauria to become the most successful and long-lived radiation of marine reptiles [15, see also 59]. Large body size, high growth rates and presumably high basal metabolic rates must have facilitated the pelagic lifestyle of adult plesiosaurs (the juveniles apparently having inhabited coastal waters [38]) and rapid spread of plesiosaurs around the globe since the Early Jurassic.

This hypothesis raises the issue of the lack of a Triassic record of the Plesiosauria because Plesiosauria are separated from Pistosauridae by a 30 million years ghost lineage [15]. There are two possible, not mutually exclusive, explanations: Triassic Plesiosauria may have inhabited the Southern Hemisphere and only spread to the Northern Hemisphere in the Jurassic, or the remains of Triassic Plesiosauria have not been discovered yet because of the paucity of Late Triassic marine reptile faunas. The latter view is supported by the extremely long duration of the Norian stage of about 25 million years, which only recently has been recognized (e.g., [61]). This makes it by far the longest stage of the entire Phanerozoic, but considerably fewer marine reptile *Lagerstätten* are known from the Norian than from the much shorter Middle Triassic stages.

Support for our hypothesis is also found in the paleobiogeography of Pistosauroidea. *Corosaurus* from the early Middle Triassic of Wyoming, USA [19-21] is a basal pistosauroid. Its presence on the western shelf of North America indicates that Pistosauroidea were already able to disperse across the Northern Hemisphere at this early time, unlike all other sauropterygian clades which remained restricted to the Tethys. If basal pistosauroids were already characterized by a high basal metabolic rate, this would indicate a general evolutionary pattern in the secondary adaptation to the marine environment where changes in physiology predate morphological adaptations to a pelagic lifestyle.

Our results support the hypothesis of Motani [15] and Bernard et al. [85] that a high basal metabolic rate and at least incipient endothermy is the prerequisite for the spread of a secondarily aquatic lineage across the globe. This applied even during the Mesozoic with its generally higher sea surface temperatures. Lineages that were not able to evolve a constant high basal metabolic rate remained restricted to the warm shallow seas, the Nothosauroidea studied by us being a case in point. The pattern of extinction at the end of the Middle Triassic, with Nothosauroidea disappearing from the fossil record, but Placodontia and Pistosauria surviving, may also be explained by the differences in thermophysiology as revealed by bone histology. Lineages with high basal metabolic rates may have been less affected by global sea level and temperature changes because of their ability to inhabit cooler waters and the open ocean.

## Conclusions

We studied the long bone microanatomy and histology of the stem-group sauropterygians *Nothosaurus* spp. and *Pistosaurus longaevus* from the Muschelkalk (Middle Triassic) and of the Early Jurassic plesiosaur *Plesiosaurus dolichodeirus*. *Nothosaurus* long bones generally consist of RLLZB. However, at least one juvenile nothosaur individual grew with continuously deposited FLB (MHI 633). The Lower Muschelkalk *N*. *marchicus*/*N*. *winterswijkensis* humerus, a small Upper Muschelkalk *Nothosaurus* sp. indet. humerus, and *Nothosaurus* femora all show a thick cortex (MI around 30%) of LZB, consistent with previous studies [59].

Humeri of adult Upper Muschelkalk *N*. *giganteus* and *N*. *mirabilis* show an exceptional histological pattern: a very thin cortex (MI around 70%) and a much enlarged medullary region (Figure 3). The thin bone walls combined with the increased diameter of the triangular diaphyseal cross section were well suited to resist bending loads. This suggests that in the two large-bodied *Nothosaurus* species the forelimbs played an important role in locomotion. Nothosauroidea may have evolved towards an increasingly pelagic lifestyle convergently to Pistosauria, not owing to a higher basal metabolic rate, but due to their newly achieved biomechnical adaptations, involving a lightening of the skeleton and some kind of limb-based propulsion.

In contrast to *Nothosaurus*, the *Pistosaurus* humeri show a cortex entirely formed by FLB with mainly radial vascular canals and have a very small marrow cavity. *Pistosaurus* histology thus reflects high growth rates but gives no indication that the forelimb was important in propulsion, unlike in the later plesiosaurs. The femur of *Plesiosaurus dolichodeirus* from the Lower Jurassic of Lyme Regis, UK, also consists of FLB. However, unlike any of the Triassic sauropterygians, it was intensively remodeled (compare with [38]).

FLB, and by extension high growth rates and high basal metabolic rates, had already evolved among stem-group Sauropterygia by Ladinian times in the Pistosauria or even earlier [59]. The invasion of the pelagic realm by the Pistosauroidea already in the Middle Triassic correlates with a substantial increase in body size, presumably facilitated by the increased metabolic rate that had evolved in earlier Pistosauroidea and was retained in the later Pistosauria (Pistosauridae and Plesiosauria). A high basal metabolic rate may have been a prerequisite for the pelagic life style of Pistosauridae and Plesiosauria by keeping the body warm in cold waters, providing endurance and making a sufficiently large body size possible.

## Abbreviations

SIPB: Steinmann-Institut für Geologie, Mineralogie und Paläontologie, Universität Bonn, Germany; MfN: Museum für Naturkunde – Leibniz-Institut für Evolutions- und Biodiversitätsforschung an der Humboldt-Universität zu Berlin, Berlin, Germany; MHI: Muschelkalkmuseum Hagdorn, Ingelfingen, Germany; SMNS: Staatliches Museum für Naturkunde Stuttgart, Stuttgart, Germany.

## Competing interests

The authors declare that they have no competing interests.

## Authors’ contributions

AK, NK, and PMS designed research; AK and PMS performed research; AK, NK, and PMS analyzed data; and AK and PMS wrote the paper. All authors read and approved the final manuscript.

## Supplementary Material

Additional file 1 **The sample of eosauropterygian long bones Information on genus, species, bone type, stratigraphy, locality, and dimensions are given for each sampled specimen.** Humeri and femur of *Pistosaurus*, and the femur of *Plesiosaurus dolichodeirus* could not confidently be assigned to a body side because of their simplified morphology and insufficient descriptions in the literature. MHI 1906 is incomplete, and the circumference had to be measured 5 mm distally of section plane. Abbreviations: c, circumference; dl, distal length; dw, distal width; ml, measurable length; msl, minimal shaft length; msw, minimal shaft width; pl, proximal length; pw, proximal width; tl, total length; /, absent. Click here for file
